# Differentially expressed circRNAs in peripheral blood samples as potential biomarkers and therapeutic targets for acute angle-closure glaucoma

**DOI:** 10.1038/s41598-023-44073-y

**Published:** 2023-10-07

**Authors:** Ruijuan Guan, Suonan Angxiu, Ling Li, Zefeng Kang, Xin Yan

**Affiliations:** 1 Ophthalmology Department, Qinghai Provincial People’s Hospital, 2 Gonghe Road, Xining, 810000 Qinghai China; 2 Orthopedics Department, Qinghai Provincial People’s Hospital, 2 Gonghe Road, Xining, 810000 Qinghai China; 3 Eye Hospital, China Academy of Chinese Medical Sciences, 33 Lugu Road, Beijing, 100040 China

**Keywords:** Genetics, Biomarkers, Eye diseases

## Abstract

Glaucoma is the leading cause of irreversible blindness globally. Circular RNAs (circRNAs) play vital roles in various biological processes as microRNA (miRNA) sponges and, thus, have been investigated as potential biomarkers and therapeutic targets in numerous human diseases. However, the underlying mechanisms of circRNAs in the pathogenesis of glaucoma remain unclear. Therefore, transcriptome sequencing was performed to identify relevant circRNAs in peripheral blood samples from patients with primary angle-closure glaucoma. Bioinformatics analysis was performed to investigate the potential roles of differentially expressed circRNAs (DEcircRNAs) in the pathogenesis of glaucoma. In total, 481 differentially expressed genes in addition to 345 DEcircRNAs were identified in patients with glaucoma. Based on a public database, targeted gene analysis identified 11 DEcircRNAs that potentially regulate the expression of five genes as miRNA sponges in glaucoma. In addition, quantitative reverse transcription PCR analysis verified that expression of the circRNA hsa-circ-0000745 was positively correlated with the expression of NEAT1 as a potential target gene. These results suggest that DEcircRNAs are involved in a gene expression regulatory network related to immune cell function and progression of glaucoma. Thus, DEcircRNAs in peripheral blood are potential biomarkers and therapeutic targets for glaucoma.

## Introduction

Glaucoma is a collective term for a group of degenerative diseases of the optic nerve, characterized by optic nerve damage and visual field defects, and is currently the leading cause of irreversible blindness globally with a prevalence of ~ 3.5% after the age of 40 years^1^. Common features of all forms of glaucoma include progressive degeneration of the optic nerve and death of mature retinal ganglion cells (RGCs), which do not regenerate. Hence, visual damage resulting from death of RGCs is irreversible^2^. Ethnicity is related to the prevalence of different forms of glaucoma, with primary angle-closure glaucoma being the dominant type affecting Asian people^3^.

Early diagnosis and treatment are essential to prevent the progression of glaucoma. The genetic testing results of blood samples and primary or metastatic tumor specimens are generally in agreement for various diseases. Therefore, it is reasonable that similar genetic methods could be useful to identify molecular markers in blood samples for early diagnosis of glaucoma. Primary glaucoma usually affects both eyes as they share the same anatomical features. Thus, biomarkers can be particularly helpful for diagnosing glaucoma in the second eye early after a patient has had a glaucoma attack in one eye.

Circular RNAs (circRNAs) are a class of tissue- and cell-specific noncoding RNAs mainly synthesized by back-splicing of post-transcription pre-mRNAs. They perform various biological functions by mainly acting as sponges to bind microRNAs (miRNAs) or protein inhibitors to regulate protein function^4^. Numerous circRNAs have been implicated in the development of glaucoma and investigated as potential biomarkers and therapeutic targets^5,6^. For example, a mouse study of glaucoma reported that circRNAs in the retinal tissue influence the development of glaucoma by sponging miRNAs resulting in retinal nerve degeneration^7,8^. Moreover, a study of glaucoma-associated circRNAs in the retinal tissues of a chronic ocular hypertension rat model identified hsa_circ_0023826 as a potential biomarker for chronic high intraocular pressure (IOP)^9^, while an investigation of circRNAs in the retinal tissues of a mouse model of retinal ischemia–reperfusion (I/R) injury suggested Wdr37 as a potential diagnostic or therapeutic target for retinal I/R-related diseases^10^. However, relatively few studies have investigated the expression profiles of circRNAs in peripheral blood or other samples to identify potential diagnostic biomarkers for glaucoma.

Therefore, the aim of the present study was to identify circRNAs in peripheral blood samples as potential diagnostic biomarkers for primary angle-closure glaucoma. Sequencing of circular RNA (circRNA-Seq) and bioinformatics analysis were performed and a circRNA–mRNA regulatory network was constructed to examine the roles of these circRNAs in the development of glaucoma. This study identified differentially expressed circRNAs (DEcircRNAs) in peripheral blood samples as potential biomarkers and therapeutic targets for primary angle-closure glaucoma.

## Material and methods

### Study approval and patient consent

The study protocol was approved by the Ethics Committee of Qinghai Provincial People's Hospital (approval number: 2023-141) and conducted in accordance with the ethical principles for medical research involving human subjects described in the Declaration of Helsinki in addition to relevant Chinese laws and institutional guidelines. Prior to inclusion in this study, written consent was obtained from all participants after being informed of the nature and possible consequences of the experiments.

### Clinical information of patients

The study cohort consisted of 5 patients with primary angle-closure glaucoma and 5 patients with cataracts but normal IOP, optic disc size, and anterior chamber angle as a control group. All participants underwent comprehensive evaluations that included visual acuity testing, IOP measurements, slit lamp examinations, color fundus retinal imaging, and optic nerve examinations. Fundus examinations with fully dilated pupils were performed by an ophthalmologist and images were captured with a fundus camera equipped with a 90D lens. A standard questionnaire was used to collect medical history data regarding glaucoma, including the date of diagnosis and current use of medications.

The inclusion criteria were age ≥ 18 years, treatment for early to moderate glaucoma (visual field mean deviation ≥  − 10 dB) with visual acuity of at least 6/18, and reliable visual field test results (Swedish Interactive Thresholding Algorithm-Standard 24-2; Humphrey 750i Visual Field Analyzer II; Carl Zeiss Meditec AG, Jena, Germany) within the last 6 months (< 33% fixation losses, false positives, and false negatives).The VCDR > 0.5 in either eye, VCDR asymmetry ≥ 0.2^11^.The only exclusion criterion was elevated IOP due to any other cause.

### RNA extraction and quality control

Peripheral venous blood samples (5 mL) were collected from all participants into anticoagulant-coated tubes and the white blood cells were extracted using a red blood cell lysis buffer. Total RNA was extracted from the whole blood samples with TRIzol™ reagent (Thermo Fisher Scientific, Waltham, MA, USA), in accordance with the manufacturer’s protocol, and treated with RQ1 DNase (Promega Corporation, Madison, WI, USA) to remove DNA. The quality and quantity of the purified RNA were determined by measuring absorbance at 260/280 nm (A260/A280) using a NanoDrop™ One spectrophotometer (Thermo Fisher Scientific). RNA integrity was verified by 1.5% agarose gel electrophoresis. The RNA samples were frozen at − 80 °C until assayed.

### RNA sequencing (RNA-Seq)

RNA-Seq libraries were prepared from 1 μg of total RNA of each sample. Ribosomal RNAs were depleted using the Ribo-off™ rRNA depletion kit (product no. N406-01; Nanjing Vazyme Biotech Co., Ltd., Nanjing, China). Directional RNA-Seq libraries were prepared from purified RNA using the KAPA Stranded mRNA-Seq Kit for Illumina® Platforms (product no. KK8544; Kapa Biosystems, Inc., Wilmington, MA, USA)^12^. Polyadenylated mRNAs were purified and fragmented.

Fragmented mRNAs were converted into double-stranded cDNAs. Following end repair and the addition of poly(A) tails, the DNA was ligated with diluted adaptors (product no. KK8726; Kapa Biosystems, Inc.) and then fractionated to 300–500 bp. The ligated and fractionated products were amplified, purified, quantified, and stored at − 80 °C until sequencing. The cDNA strand marked with 2´-deoxyuridine, 5´-triphosphate was not amplified, allowing for strand-specific sequencing. High-throughput sequencing of the libraries (150-nt paired-end sequences) was performed with a NovaSeq 6000 system (Illumina, Inc., San Diego, CA, USA) in accordance with the manufacturer's instructions^13,14^

### Cleaning and alignment of raw RNA-Seq data

Raw reads containing more than two N bases were discarded. The adaptors and low-quality bases were trimmed from the raw sequencing reads using the FASTX-Toolkit (version 0.0.13; http://hannonlab.cshl.edu/fastx_toolkit/). Short reads (< 16 nt) were also discarded. Following clean-up, the reads were aligned to the GRch38 genome (https://www.ncbi.nlm.nih.gov/assembly/GCF_000001405.26/) using the TopHat2 alignment tool^15^ allowing for four mismatches. The gene read number and fragments per kilobase of transcript per million fragments mapped (FPKM) of uniquely mapped reads were calculated^16^.

### Prediction and expression analysis of circRNAs

For the genome-wide identification of circRNAs, reads containing adapters or poly-N (unrecognized) bases and low-quality reads were filtered from the raw sequencing reads utilizing in-house Perl scripts. The resultant clean reads were mapped to the reference genome, thereby generating a sequence alignment map file. The sequence alignment map files were used to predict circRNAs with find_circ software (version 1.2; https://github.com/marvin-jens/find_circ) and default parameters^17^. Based on genomic origins, the circRNAs were classified into three major types: exonic, intronic, and intergenic. For quantification of circRNAs^18^, the number of back-spliced reads of each circRNA was normalized to the total sequencing reads in a corresponding sample data set and defined as reads per million mapped reads.

### Analysis of differentially expressed genes (DEGs) and differentially expressed circRNAs (DEcircRNAs)

DEGs were screened with the Bioconductor package edgeR^19^ with the cut-off criteria of *p*-value < 0.01 and fold change (FC) > 1.5 or < 2/3. DEGs and DEcircRNAs between the case group and control group were analyzed using the R package “TCC” (an acronym for Tag Count Comparison)^20^. For each gene, the *p*-value and false discovery rate (FDR) were obtained based on the negative binomial distribution model. FCs of gene expression were estimated with the edgeR statistical package. The only criteria for DEcircRNAs were FC > 2 and FDR < 0.05.

### Functional enrichment analysis

Gene ontology (GO) terms and enriched pathways from the Kyoto Encyclopedia of Genes and Genomes were assigned to the DEGs to clarify the functional categories using the KOBAS 2.0 server^21^. The hypergeometric test and Benjamini–Hochberg FDR controlling procedure were used to define the enrichment of each term.

### CeRNA network of DEGs with DEcircRNAs and miRNAs

The miRanda database (https://anaconda.org/bioconda/miranda) was searched to identify potential complementary pairs of miRNAs and circRNAs. All miRNA–circRNA pairings with a miRanda score ≥ 150 were retained. The rnahybrid tool (https://bibiserv.cebitec.uni-bielefeld.de/rnahybrid/) was also used to identify potential miRNA–circRNA pairs, and all miRNA–circRNA pairs with *p*-values ≤ 0.05 were retained. Finally, the results of the two methods were compared to obtain miRNA–circRNA pairs identified by both methods as the final miRNA-circRNA target relationship.

The results of queries of the miRDB online database for prediction of functional miRNA targets (http://mirdb.org) and the TargetScan web server to predict biological targets of miRNAs (http://www.targetscan.org) were combined to identify the target DEGs of the differentially expressed miRNAs.

### Validation of DEGs and DEcircRNAs

Quantitative reverse transcription PCR (RT-qPCR) analysis of some of the identified DEGs and DEcircRNAs was performed to assess the validity of the RNA-Seq data. The primers used for RT-qPCR analysis are listed in Supplementary Table S1. Total RNA remaining from preparation of the RNA-Seq libraries was used for RT-qPCR analysis. Three technical replicates were used for the amplification of each DEG and DEcircRNA of each sample. The expression levels of all genes were normalized against expression of glyceraldehyde-3-phosphate dehydrogenase (GAPDH) as a reference gene using the 2^−ΔΔCt^ method^22^.

### Statistical analyses

All statistical analyses were performed using IBM SPSS Statistics for Windows, version 20.0. (IBM Corporation, Armonk, NY, USA) and graphs were generated with GraphPad Prism 5 software (GraphPad Software, Inc., San Diego, CA, USA). Continuous variables are presented as the mean ± standard deviation. The Student’s *t*-test or Mann–Whitney test was used as appropriate for comparisons of two groups of independent samples. Fisher’s exact test or the chi-square test was employed for analysis of categorical variables.

## Results

### Basic clinical characteristics of the study participants

The patient and control groups were well matched in regard to age and sex. There were five cases of acute angle-closure glaucoma and five cases of cataracts. The basic characteristics of all participants are summarized in Table 1.

**Table 1:** Basic clinical characteristics of all participants.

Characteristic	Patient group	Control group	*X*^*2*^*/t*	*p*
Age, years	64.20 ± 4.87	64.40 ± 5.41	0.061	0.953
Intraocular pressure, mmHg	48.20 ± 17.70	13.60 ± 1.82	4.475	0.011
Optic disc size	0.68 ± 1.30	0.44 ± 0.55	3.795	0.005
Peripheral anterior chamber depth,CT	1.40 ± 0.22	2.40 ± 0.55	3.780	0.012

### RNA-Seq analysis of blood samples collected from patients with glaucoma

**Figure 1:**
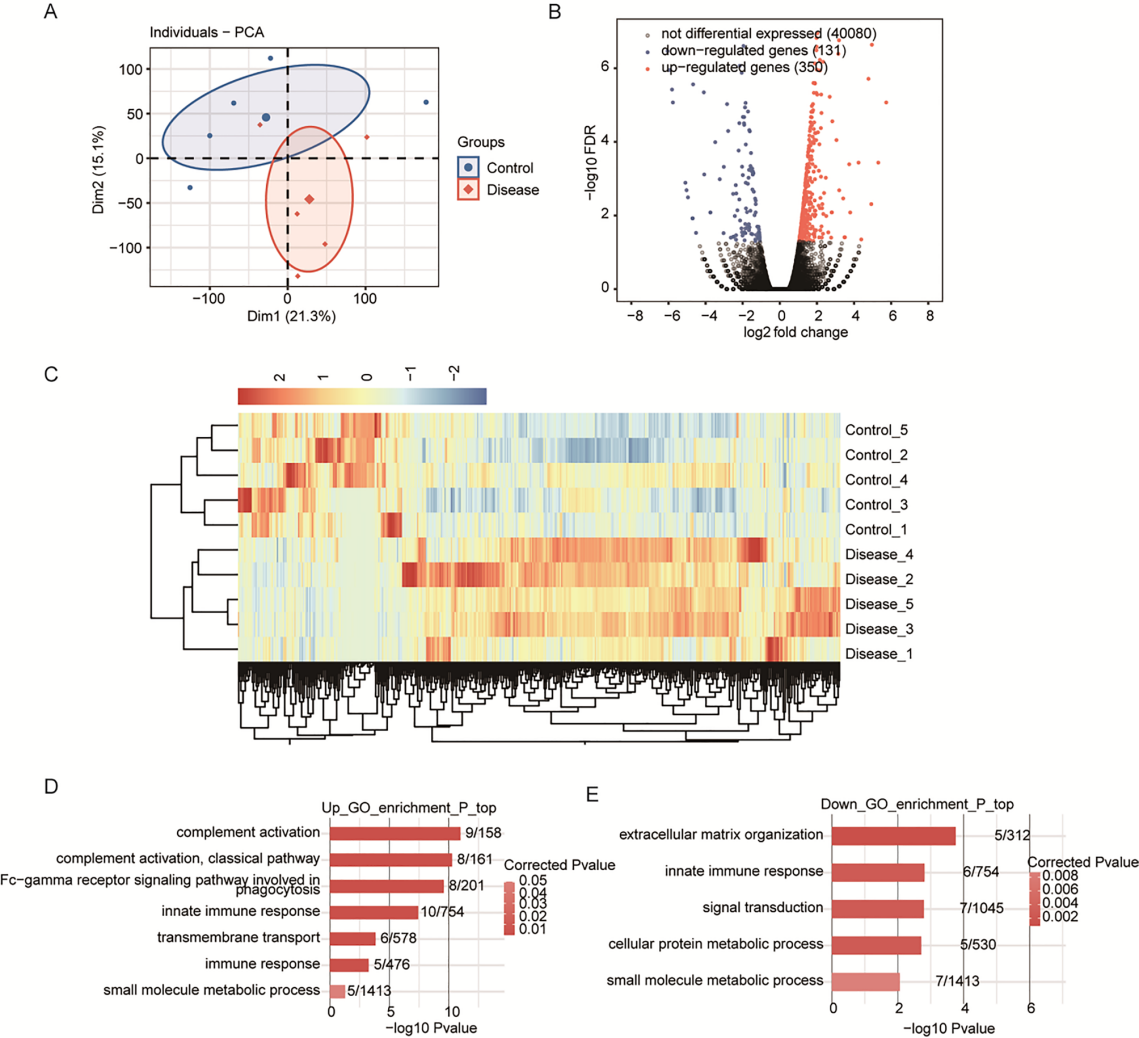
(**A**) Principal component analysis based on gene expression in glaucoma and control samples. (**B**) Identification of differentially expressed genes (DEGs) between the patient and control groups. Volcano plot shows upregulated and downregulated genes in red and blue, respectively. (**C**) Hierarchical clustering of DEGs in the patient and control groups. The median log2(fragments per kilobase of transcript per million fragments mapped) value was calculated for each gene. (**D**) The top 10 Gene Ontology (GO) biological processes of upregulated genes. (**E**) The top 10 GO biological processes of downregulated genes.

Transcriptome sequencing analysis was performed to identify DEGs associated with glaucoma. Correlation coefficients among the transcriptome sequencing data of the samples per group were calculated and cluster analysis was performed. As shown in the cluster plot presented in Fig. 1A, the glaucoma and control samples generally clustered into separate groups with the exception of one sample of each group, indicating differences in the gene expression profiles of blood samples between the patient and control groups, as well as heterogeneity among the participants. In total, 481 DEGs (350 upregulated and 131 downregulated) between the patient and control groups met the criteria of FC = 1.5 and *p* < 0.01 (Fig. 1B,C). GO analysis revealed functional pathways the DEGs were enriched in (Fig. 1D,E). Notably, complement and immune-related genes (e.g., CFD, IGHG1, and IGHA1) were enriched. In addition, SNORD3A, SNORD16, SNORA52, and SNORA23 were differentially expressed in the patient group, thereby warranting further analyses.The full names of the genes are listed in Supplementary Table S2.

### Identification and characteristics of glaucoma-related circRNAs as potential biomarkers

**Figure 2:**
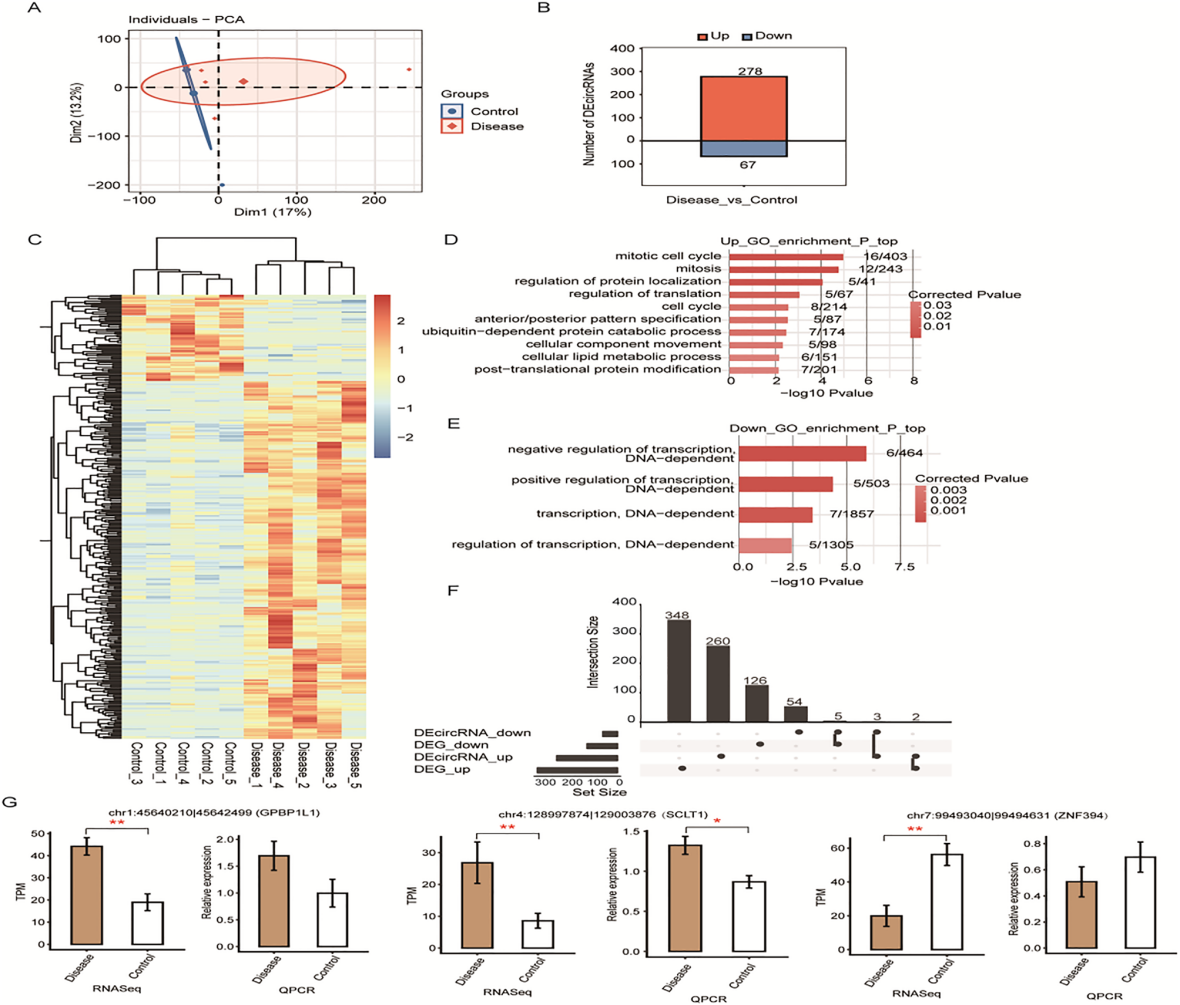
(**A**) Principal component analysis based on circRNA expression in glaucoma and control samples. (**B**) Number of differentially expressed circRNAs (DEcircRNAs) between the patient and control groups. (**C**) Hierarchical clustering of DEcircRNAs. The median log2(transcript per million) value was calculated for each gene. (D) The top 10 Gene Ontology (GO) biological processes of the host genes of upregulated circRNAs. (**E**) The top 10 GO biological processes of host genes of downregulated circRNAs. (**F**) Overlap analysis between host genes of DEcircRNAs and differentially expressed genes. The black dots indicate the gene groups for overlap analysis. The bars with numbers indicate the number of genes in each group or overlapped groups. (**G**) Bar plots showing the expression pattern and statistical difference of selected DEcircRNAs determined by RNA-Seq (left) and RT-qPCR (right) analyses of the patient and control groups. Error bars represent the mean ± SD. GAPDH was used as a reference gene for RT-qPCR analysis. The expression levels of each gene in the patient and control groups were compared with the Student’s t-test (*p < 0.05, **p < 0.01, ***p < 0.001).

Differences in circRNA sequencing data between the patient and control groups were identified using findcirc software (v1.2; https://github.com/marvin-jens/find_circ). As shown in Fig. 2A, the glaucoma and control samples formed separate clusters. In total, 345 DEcircRNAs (278 upregulated and 67 downregulated) were identified in the patient group (Fig. 2B,C). Bioinformatics analysis was conducted to identify the host genes of the DEcircRNAs, and GO analysis was performed to investigate the functions of these host genes. The results showed that the host genes of the upregulated circRNAs were mainly enriched in the GO terms “mitotic cell cycle,” “mitosis,” “regulation of protein localization,” “translational regulation,” “cell cycle,” “ubiquitin-dependent protein catabolism processes,” “cellular lipid metabolism processes,” and “post-translational protein modification” (Fig. 2D). The host genes of the downregulated circRNAs were mainly enriched in the terms “DNA-dependent positive transcriptional regulation,” “DNA-dependent negative regulation,” “DNA-dependent transcription,” and “DNA-dependent transcriptional regulation” (Fig. 2E). Furthermore, seven host genes (NEAT1, SAMD12, KDM5D, ZFY, EIF1AY, TXLNGY, and DDX3Y) of nine DEcircRNAs (chr11:65435205/65435362, hsa_circ_0135571, hsa_circ_0140781, hsa_circ_0001953, hsa_circ_0007907, hsa_circ_0140784, hsa_circ_0009024, hsa_circ_ 0140779, hsa_circ_0005757) were also among the DEGs identified (Fig. 2F). The trend of differential expression of these circRNAs and their host genes was consistent (Fig. 2G).

### Validation of glaucoma-associated DEGs

**Figure 3:**
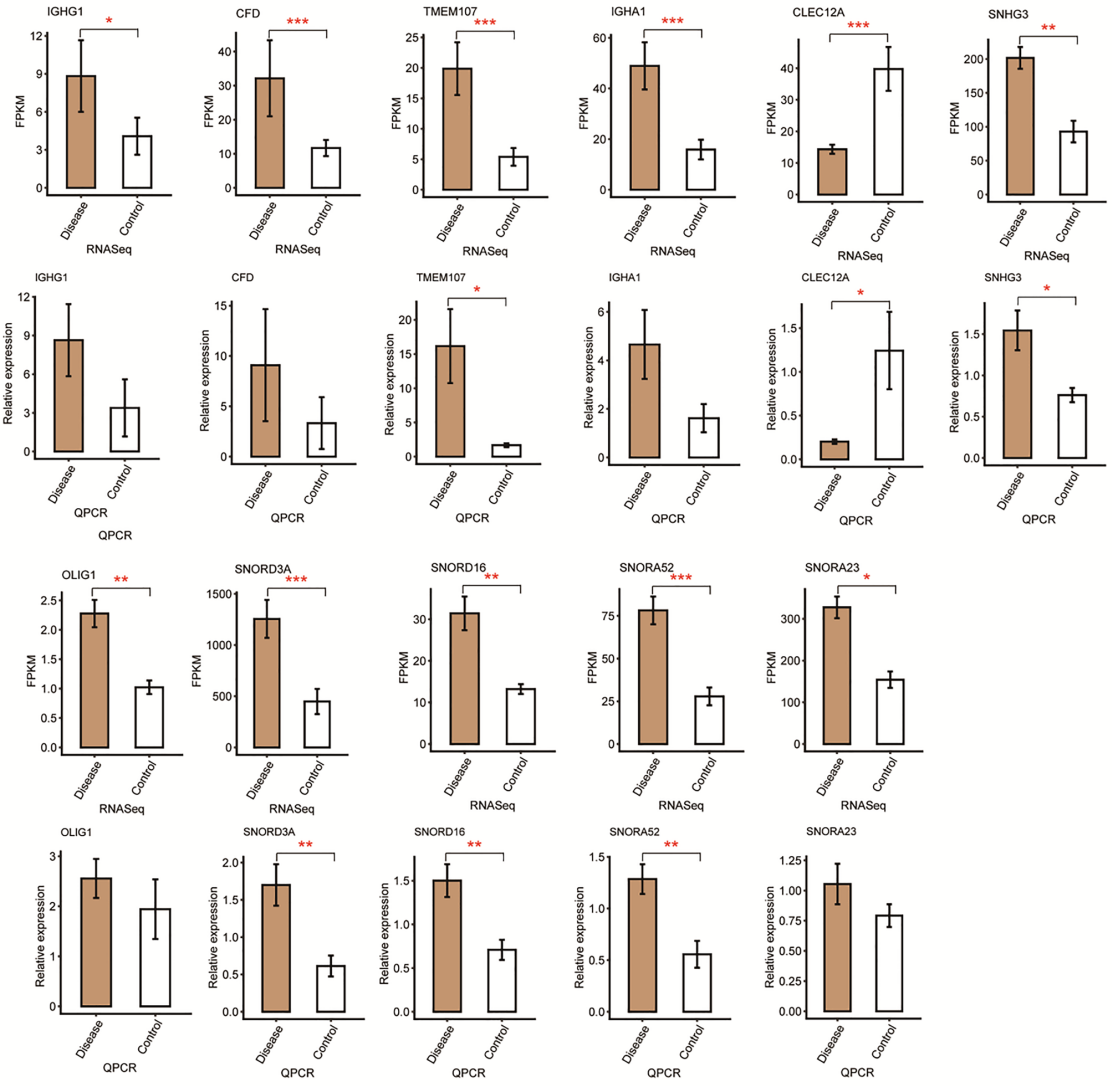
Bar plots showing the expression patterns and statistical differences of the selected differentially expressed genes (DEGs) determined by RNA-Seq (upper rows) and RT-qPCR (lower rows) analyses of the patient and control groups. Error bars represent mean ± standard error of the mean (SEM). GAPDH was used as a reference gene for RT-qPCR analysis. The expression levels of each gene in the patient and control groups were compared with the Student’s *t*-test (*p < 0.05, **p < 0.01, ***p < 0.001).

The DEGs presumptively associated with glaucoma were validated by qRT-PCR analysis. The results showed that IGHG1, CFD, IGHA1, TMEM107, SNHG3, OLIG1, SNORD3A, SNORD16, SNORA52, and SNORA23 were significantly upregulated, while CLEC12A was significantly downregulated in the patient group. These findings were consistent with the results of RNA-Seq (Fig. 3).

### Co-expression network of DEcircRNAs and DEGs associated with glaucoma

**Figure 4:**
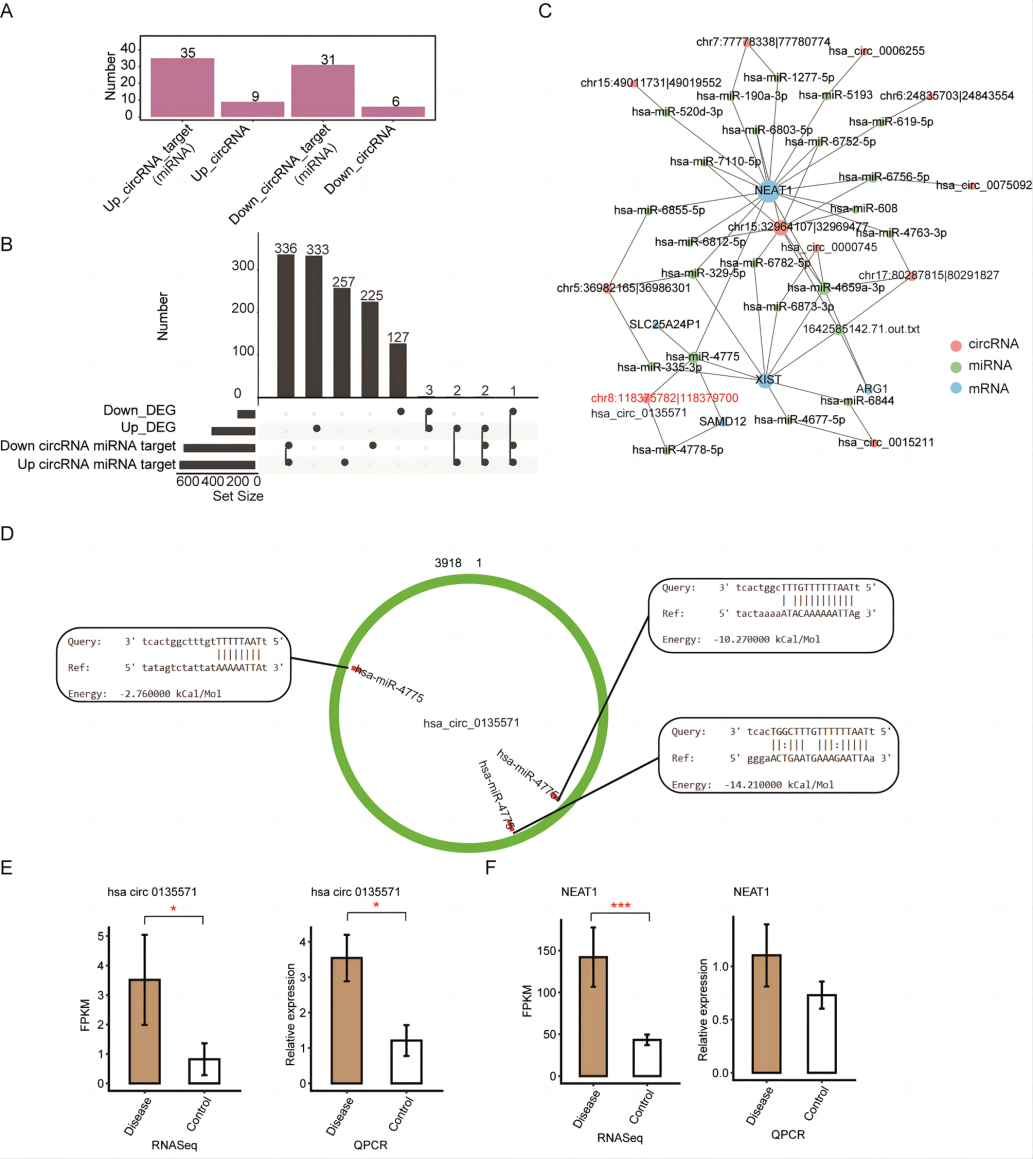
Construction of a circRNA–miRNA–mRNA network. (**A**) Number of miRNA targets of up- and downregulated differentially expressed circRNAs (DEcircRNAs) in the patient group. The miRNA targets of DEcircRNAs were obtained from the miRanda database. (**B**) Overlap analysis between the mRNA targets of miRNAs that are targeted by DEcircRNAs and differentially expressed genes (DEGs). The black dots indicate the gene groups of the overlap analysis. The bars with numbers indicate the number of genes of each group or overlapped group. (**C**) Network of DEGs, DEcircRNAs, and miRNAs. (**D**) Binding site of hsa-miR-4475 on circRNA hsa-circ_0135571. (**E**) Bar plots showing the expression pattern and statistical difference of has-circ_0135571 determined by RNA-Seq (left) and RT-qPCR (right) analyses of the patient and control groups. (**F**) Bar plots showing the expression pattern and statistical difference of NEAT1 determined by RNA-Seq (left) and RT-qPCR (right) analyses of the patient and control groups. Error bars represent mean ± SEM. GAPDH was used as a reference gene for RT-qPCR analysis. The expression levels of each gene in the patient and control groups were compared with the Student’s *t*-test (*p < 0.05, **p < 0.01, ***p < 0.001).

Numerous studies have shown that circRNAs can adsorb miRNAs to regulate expression of target genes. Potential binding pairs of miRNAs and DEcircRNAs were retrieved from the miRanda database to determine whether the DEcircRNAs may have contributed to the differential expression of DEGs we identified. The results showed that nine upregulated and six downregulated DEcircRNAs potentially bind to 35 and 31 miRNAs, respectively (Fig. 4A). Moreover, these DEcircRNAs and miRNAs could potentially form 67 interacting pairs (miRanda score > 500). Further analysis with the miRanda database revealed that these 64 miRNAs could target 823 genes. Overlap analysis of the 823 genes and 481 DEGs identified 5 DEGs as potential targets of these miRNAs (Fig. 4B). These results indicate that the DEcircRNAs could participate in the regulation of DEGs associated with glaucoma.

A binding-target network of DEcircRNAs–miRNAs–DEGs identified 11 DEcircRNAs that could potentially regulate the expression of 5 DEGs (NEAT1, XIST, ARG1, SAMD12, and SLC25A24P1) through 23 miRNAs. Notably, NEAT1 was identified as a primary target of significant proportions of these miRNAs and DEcircRNAs (Fig. 4C). For example, hsa_circ_0135571 may regulate the expression of NEAT1 by binding to hsa-miR-4775. In addition, binding site analysis showed that hsa_circ_0135571 has three potential binding sites for hsa-miR-4775 (Fig. 4D).

Lastly, RT-qPCR analysis was conducted to validate the expression patterns of hsa_circ_0135571 and NEAT1 in blood sample of the patient and control groups. The results showed that the expression levels of both hsa_circ_0135571 and NEAT1 were significantly upregulated in the patient group as compared with the control group (Fig. 4E–F). These results suggest that hsa_circ_0135571 may regulate expression of NEAT1 though sponging of miRNAs.

## Discussion

Glaucoma is characterized by optic nerve damage and visual field defects. The lack of targeted biomarkers adversely affects the quality of life of patients with glaucoma. In this study, high-throughput sequencing identified 481 DEGs in whole blood samples of glaucoma patients as compared with healthy controls. GO functional enrichment analysis revealed that 350 upregulated DEGs were enriched in functional pathways such as the classical complement activation pathway, immune response, and small molecule metabolic processes, while 131 downregulated DEGs were enriched in functional pathways such as extracellular matrix organization, innate immune response, and signal transduction. Among these, the complement- and immune-related genes CFD, IGHG1, and IGHA1 were significantly upregulated. Additionally, SNORD3A, SNORD16, SNORA52, and SNORA23 were differentially expressed in patients with glaucoma.

Aberrantly expressed circRNAs in blood are potential biomarkers and therapeutic targets for a variety of human diseases^23^. Analysis of the circRNA sequencing data with findcirc software identified 345 DEcircRNAs (278 upregulated and 67 downregulated) as potential biomarkers of glaucoma. Database analysis revealed that seven host genes (NEAT1, SAMD12, KDM5D, ZFY, EIF1AY, TXLNGY, and DDX3Y) of nine DEcircRNAs were also differentially expressed in blood samples of patients with glaucoma, and the trend of differential expression of circRNAs and host genes was consistent.

GO analysis revealed that the DEcircRNAs are involved in the development of glaucoma mainly through regulation of the complement activation pathway and immune responses. The complement system is a major component of the innate immune response and plays a crucial role as the first line of defense against invading microorganisms, clearance of apoptotic cells, and regulation of the adaptive immune response^24^. Although relatively few studies have investigated the role of the complement system in the pathogenesis of glaucoma, interactions between the complement system and oxidative stress have been established. A previous study reported that complement factors, either from local or systemic sources, protect against oxidative stress in cells and that supplementation of exogenous complement factor proteins reduced cell death, complement activation, and membrane and mitochondrial damage^25^.

However, few studies have investigated circRNAs in blood samples as potential biomarkers of glaucoma. Although the biological functions and regulatory mechanisms remain unclear, hsa_circ_0001953 and hsa_circ_0009024 were differentially expressed in peripheral blood samples of patients with glaucoma. CircRNAs can function as miRNA sponges or as competitive endogenous RNAs to compete with other RNAs for pairing to miRNAs^26,26^. CircRNAs can also regulate transcription by binding to transcription factors in the nucleus^27^. CircRNAs are more abundant in the nervous system than in other tissues^28^, and more stable in tissues, longer lived, and more resistant to RNase than miRNAs^29^. Various circRNAs have been implicated in the pathogenesis of neurodegenerative diseases^30^. Most circRNAs act as molecular sponges that bind to miRNAs via multiple binding sites, thereby inhibiting binding of the miRNAs to their target genes^31^. A recent study^32^ suggested that circRNAs play important roles in the pathogenesis of glaucoma, although the underlying mechanisms remain unclear. In the present study, hsa_circ_0001953 and hsa_circ_0009024 were differentially expressed in blood samples collected from patients with glaucoma. Analysis of a circRNA–miRNA–mRNA interaction network found that hsa_circ_0000745 and DEcircRNAs could potentially form a regulatory relationship with ARG1 and NEAT1 through the miRNAs, which may affect immune cell function, thereby promoting the development of glaucoma. Downregulation of ARG1, a member of the arginase family, has been associated with hyperarginemia and subsequent neurological impairment^33^.The mechanisms underlying upregulation of NEAT1 in human malignancies are complex and involve multiple factors, including the adsorption of tumor-suppressive miRNAs^3^. Notably, upregulation of NEAT1 is an independent marker of poor prognosis of human malignancies. Although the specific roles of NEAT1 in glaucoma and the nervous system remain unclear, the results of the present study offer useful information for future explorations of the role of hsa_circ_0000745 in the pathogenesis of glaucoma.

There were some limitations to the present study that should be addressed. First, the sample size was small and all participants were from the same region, which may have biased the results. Second, quantification of circRNAs could be influenced by the methods used for sample preparation and analysis. Therefore, large multicenter studies are needed to validate these results and assess the impacts of multiple covariates.

## Conclusions

In summary, we identified glaucoma-related DEGs and DEcircRNAs. Analysis of a circRNA–miRNA–mRNA interaction network found that aberrant expression of hsa_circ_0000745 in patients with glaucoma may regulate NEAT1 expression through hsa-miR-4659a-3p and hsa-miR-6873-3p. Hence, hsa_circ_0000745 is a potential biomarker for the early diagnosis of glaucoma. However, these results should be interpreted with caution, as a larger study is needed to further verify the reliability and reproducibility of circRNAs, such as hsa_circ_0000745, as biomarkers for the diagnosis and treatment of glaucoma.

### Supplementary Information

Supplementary Information.

## Data Availability

The sequencing data generated in this study are registered with the GEO database (GEO ID GSE210783).
