# Supplementary material for: Differentially expressed circRNAs in peripheral blood samples as potential biomarkers and therapeutic targets for acute angle-closure glaucoma
Source: Sci Rep. 2023 Oct 7;13:16928. doi: 10.1038/s41598-023-44073-y (PMC10560268; doi:10.1038/s41598-023-44073-y)
Supplement: Supplementary file 1 — Supplementary Information. [file 41598_2023_44073_MOESM1_ESM.pdf]

Table S1： The primers of each DEGs and for DEcircRNAs for RT-qPCR

CLEC12A-F	GAGGCATGAGGCATCAAT
CLEC12A-R	TGGCATTAACATTCACAGTC
TMEM107-F	AAGAATCAGACAGGAGCAAT
TMEM107-R	CGGAGGGCAGATTAGAAC
OLIG1-F	CACCTTTCGTTTCCCTTTC
OLIG1-R	TCCTGCGTGTTAATGAGAA
SNORD16-F	TTCGTCAACCTTCTGTACC
SNORD16-R	GCGTCTTACTCTGTTCTCA
SNORA52-F	TTCGCTTGTGGACCAGAG
SNORA52-R	AGTGTCTAGAAGTGCCCAT
SNORA23-F	GCAGTGTCTGTCTGTGTT
SNORA23-R	GCCAGTGGTAGATGTGTC
IGHA1-F	TGACAACAGACACATTGACA
IGHA1-R	TACCTGACTTGGGCATCC
SNHG3-F	TCTGTCCAAGTGGCGTAG
SNHG3-R	TCTGGGAAGCTGTAGGAATA
NEAT1-F	CAGTGTGAGTCCTAGCATT
NEAT1-R	GAACTTCCTCCTCCTAAGC
SNORD3A -F	AACCACGAGGAAGAGAGG
SNORD3A -R	GAGAAGAACGATCATCAATGG
hsa_circ_ HDAC5-F	GCACTCTGAATACCACACC
hsa_circ_ HDAC5-R	TCCTCCTCTGTCTCCTCA
hsa_circ_ SAMD12-F	ACAGTTACTCACACAAGGAA
hsa_circ_ SAMD12-R	TTGTCAGTAAGTCTCAGCAG
hsa_circ_ PAK1IP1-F	GATGTGCCAGTCAAGTTG
hsa_circ_ PAK1IP1-R	CCAGATACAGATGAGTCCAT
hsa_circ_ ZNF789-F	AGTGAGTGAGGAATGAGGA
hsa_circ_ ZNF789-R	GGAACGCTTCTTGTAGTTG
hsa_circ_ RNF213-F	TACAAGCACCAGCAGAAG

hsa_circ_ RNF213-R	GATGATGGCGTGGAAGAA
hsa_circ_ GPBP1L1-F	ATTGTCATCCGTGGTTCC
hsa_circ_ GPBP1L1-R	CCATACTCCAGAAGGTGTC
hsa_circ_ DYRK1A-F	CTATACCAAGTAGATGCTCACT
hsa_circ_ DYRK1A-R	GCCTTCACACTGCTTCTT
hsa_circ_ SECISBP2L -F	GATGACTTGCCACAGGAG
hsa_circ_ SECISBP2L -R	CGCTTCTATGGGATTGACTA
hsa_circ_ FMN1 -F	CTCCTTAGAAGAACCTGACAT
hsa_circ_ FMN1 -R	GTCTCGCTGTTATCATTGC
hsa_circ_ SCLT1-F	CAACAACTGACCAAACAACT
hsa_circ_ SCLT1-R	TCTTGAAGGTTTCTGACTGT

Table S2: Full gene name

**Gene**	**Full gene name**
NEAT1	nuclear paraspeckle assembly transcript 1
SAMD12	sterile alpha motif domain containing 12
KDM5D	lysine demethylase 5D
ZFY	zinc finger protein Y-linked
EIF1AY	eukaryotic translation initiation factor 1A Y-linked
TXLNGY	taxilin gamma pseudogene, Y-linked
DDX3Y	DEAD-box helicase 3 Y-linked
IGHG1	immunoglobulin heavy constant gamma 1
CFD	complement factor D
IGHA1	immunoglobulin heavy constant alpha 1
TMEM107	transmembrane protein 107
SNHG3	small nucleolar RNA host gene 3
OLIG1	oligodendrocyte transcription factor 1
SNORD3A	small nucleolar RNA, C/D box 3A
SNORD16	small nucleolar RNA, C/D box 16

SNORA52	small nucleolar RNA, H/ACA box 52
SNORA23	small nucleolar RNA, H/ACA box 23
XIST	X inactive specific transcript
ARG1	arginase 1
SLC25A24P1	SLC25A24 pseudogene 1
CLEC12A	C-type lectin domain family 12 member A
hsa-miR-4775	hsa-miR-4775
hsa_circ_0135571	hsa_circ_0135571
hsa_circ_0140781	hsa_circ_0140781
hsa_circ_0001953	hsa_circ_0001953
hsa_circ_0007907	hsa_circ_0007907
hsa_circ_0140784	hsa_circ_0140784
hsa_circ_0009024	hsa_circ_0009024
hsa_circ_ 0140779	hsa_circ_ 0140779
hsa_circ_0005757	hsa_circ_0005757
